# Double-standards in reporting of risk and responsibility for sexual health: a qualitative content analysis of negatively toned UK newsprint articles

**DOI:** 10.1186/1471-2458-14-792

**Published:** 2014-08-04

**Authors:** Susan P Martin, Lisa M McDaid, Shona Hilton

**Affiliations:** Medical Research Council/Chief Scientist Office, Social and Public Health Sciences Unit, University of Glasgow, 200 Renfield Street, Glasgow, G2 3QB Scotland

**Keywords:** Sexual health, Media analysis, Gender, Sexual risk, Prevention

## Abstract

**Background:**

The need to challenge messages that reinforce harmful negative discourses around sexual risk and responsibility is a priority in improving sexual health. The mass media are an important source of information regularly alerting, updating and influencing public opinions and the way in which sexual health issues are framed may play a crucial role in shaping expectations of who is responsible for sexual health risks and healthy sexual practices.

**Methods:**

We conducted an in-depth, qualitative analysis of 85 negatively toned newspaper articles reporting on sexual health topics to examine how risk and responsibility have been framed within these in relation to gender. Articles published in 2010 in seven UK and three Scottish national newspapers were included. A latent content analysis approach was taken, focusing on interpreting the underlying meaning of text.

**Results:**

A key theme in the articles was men being framed as a risk to women’s sexual health, whilst it was part of a women’s role to “resist” men’s advances. Such discourses tended to portray a power imbalance in sexual relationships between women and men. A number of articles argued that it was women who needed to take more responsibility for sexual health. Articles repeatedly suggested that women and teenage girls in particular, lacked the skills and confidence to negotiate safer sex and sex education programmes were often presented as having failed. Men were frequently portrayed as being more promiscuous and engaging in more risky sexual health behaviours than women, yet just one article drew attention to the lack of focus on male responsibility for sexual health. Gay men were used as a bench mark against which rates were measured and framed as being *a* risk and *at* risk.

**Conclusions:**

The framing of men as a risk to women, whilst women are presented at the same time as responsible for patrolling sexual encounters, organising contraception and preventing sexual ill health reinforces gender stereotypes and undermines efforts to promote a collective responsibility for sexual health. This has implications for sexual ill health prevention and could continue to reinforce a negative culture around sex, relationships and sexual health in the UK.

## Background

Improving sexual health and blood borne virus (BBV) outcomes continue to be a high priority for the UK and Scottish Governments, with sexual ill health remaining an important issue to tackle [1, 2]. The Scottish Government’s Sexual Health and Blood Borne Virus Framework (2011–15) aims to encourage positive sexual health and wellbeing, and identifies a need to challenge messages that reinforce harmful negative discourses around sexual risk and responsibility [2]. The mass media are an important source of health information regularly alerting, updating and influencing public opinions and understandings on a range of health issues and risks [3, 4]. The media have also been identified as playing an important role in setting the agenda, by selecting, controlling, and prioritising media message content [5]. Through selectively emphasising particular issues, the media can shape what views and interpretations are seen to be most valid, shaping people’s perceptions of reality, risk and blame which, in turn, may influence their health behaviours [6–8]. Thus, the way in which sexual health and BBV issues are framed within the media may play a crucial role in shaping people’s understandings about sexual health risks and expectations about behaviours, setting the agenda for who is held responsible for controlling and reducing those risks or managing healthy sexual practices.

Previous research has suggested that the media are an important source of sexual health information and help to set societal ‘norms’ around sexual behaviour expectations, particularly for adolescents [9, 10]. Studies have found that more frequent exposure to a variety of sexual content in the media during adolescence, from overt portrayals of sexual behaviour (including physical flirting, touching and kissing and sexual intercourse) and depictions of risk and safety behaviours (use/non use of condoms and other forms of contraception, abstinence) to implied sexual behaviours and discussions about sexual issues, may predict earlier initiation of sexual intercourse [11, 12]. One explanation for this ‘copycat’ behaviour, based on social cognitive theory, suggests that individuals may copy the behaviour of someone they see in real-life or in the media, particularly if that person is rewarded for behaving in a particular way [13]. Thus, individuals can learn how to perform certain behaviours from media models that provide them with ideas for their own sexual behaviours [14]. Whilst media audiences may actively construct and de-construct these messages [15], it is also possible that the media may influence some adolescent understandings of appropriate and inappropriate behaviour [16] and through constant re-messaging overtime [17] create social norms around unhelpful sexual health messages [15].

Previous studies highlight the failure of mass media to promote sexually healthy behaviours [18, 19]. Safer sex messages were rarely present and when they were, they tended to focus on contraception, with little focus on other aspects of sexual health [20, 21]. It has been argued that such coverage, focusing on benefits, with little attention paid to negative consequences and risks may result in people being misinformed and could result in potentially dangerous decisions about sex [21].

A number of studies have also highlighted the way in which gender roles are portrayed within media coverage of sexual health issues, drawing attention to gender biases in framing and reinforcing existing stereotypes concerning women in relation to behaviours and responsibility [15, 22–25]. By reinforcing traditional gender stereotypes of males as sexually ‘obsessed’ and females as responsible for resisting sexual advances and for preventing the consequences (i.e. pregnancy), such messages may act to reinforce harmful behaviours and attitudes amongst men and women. Indeed these findings resonate with our previous paper, which provided an overview of UK Press reporting on sexual health and BBV topics in 2010 [26]. One issue that was highlighted from this earlier analysis warranting further investigation was in relation to a gender imbalance in news reporting about sexual health (for example, 71% of reproductive health articles focused on women and only 23% contained any mention of men) [26]. This current paper offers an in-depth, qualitative analysis of negatively toned UK newsprint articles reporting of sexual health topics to examine how risk and responsibility have been framed within these in relation to gender. It is anticipated that this will provide useful insights for public health advocates in informing their future communication strategies.

## Methods

Seven UK and three Scottish national newspapers were selected (including their corresponding Sunday editions) with high circulation figures and included a range of genres: ‘serious’, ‘middle market’ tabloid and ‘tabloid’ newspapers (Table 1). ‘Serious’ newspapers tend to be more serious in tone, politically diverse and have mostly middle-class readerships, while ‘middle market’ tabloids are comparatively less serious, attracting older, middle-class, right wing readers and tabloid newspapers are also less serious, tend to be more sensationalist, politically diverse and have mostly working-class readership. This typology has been used in other analyses of print media discourses to select a broad sample of newspapers with a range of readership profiles [15, 27].

**Table 1:** **Summary of articles (N = 85)**

Genre	Title	Total articles	
		***n***	%
**Serious**	Guardian & Observer	9	10.6
	Daily Telegraph & Sunday Telegraph	6	7.1
	Independent & Independent on Sunday	1	1.2
	Herald & Sunday Herald	4	4.7
	Scotsman & Scotland on Sunday	4	4.7
**Sub-total**		**24**	**28.2**
**Tabloid**	The Mirror & Sunday Mirror	6	7.1
	The Sun & News of the World	9	10.6
	Daily Record & Sunday Mail	6	7.1
**Sub-total**		**21**	**24.7**
**Mid-market**	Daily Mail & Mail on Sunday	18	21.2
	Daily Express & Sunday Express	17	20.0
	Evening News	4	4.7
	Evening Times	1	1.2
**Sub-total**		**40**	**47.1**
**Total**		**85**	**100**

**Figure 1:**
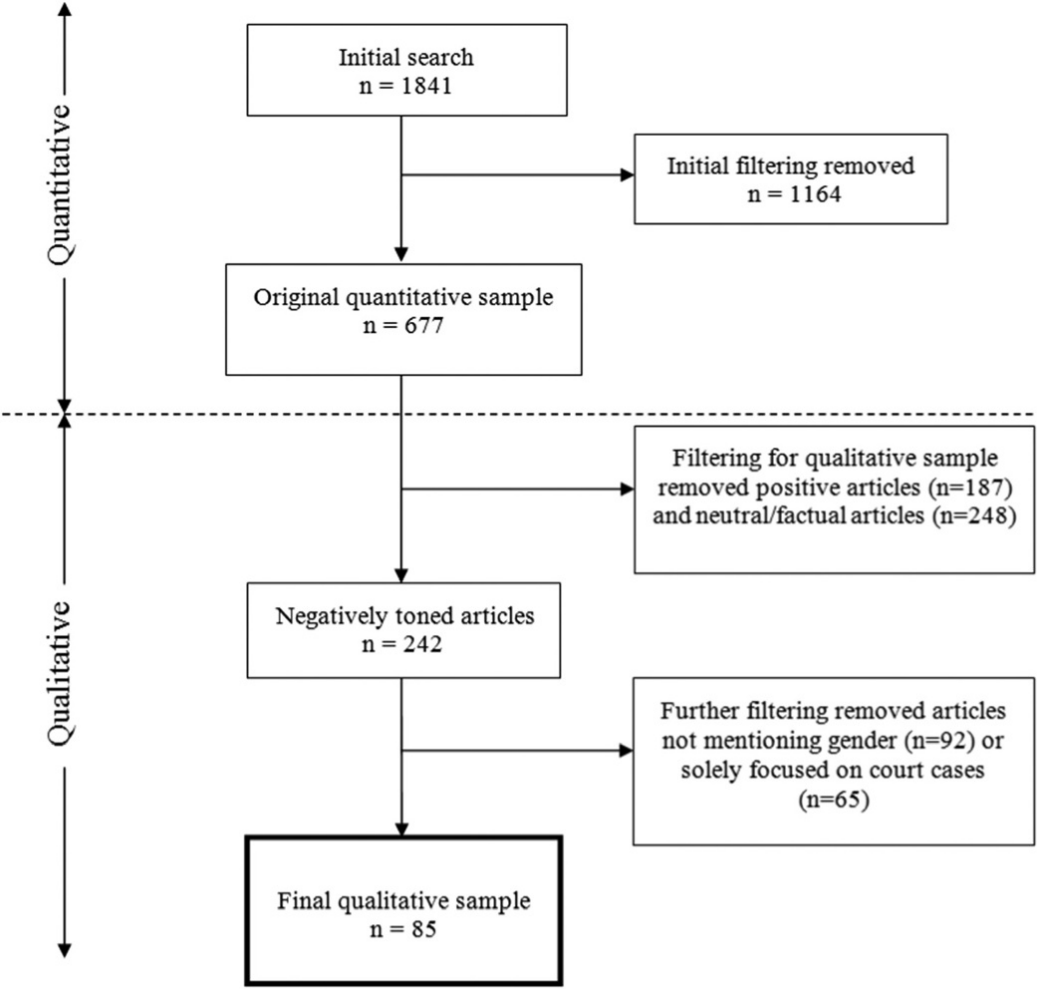
**Filtering newspaper sample.**

Our search period was from 1st January 2010 to 31st December 2010. To identify articles, electronic databases *Nexis UK* and *Newsbank* were used. They provide online access to full-text newspaper articles from over 1000 UK and Irish titles and the files can be searched for specific words or word combinations to identify articles on a specific topic. The use of such databases cannot guarantee that all articles will be identified, however, a broad range of search terms were used within full-text searches to try and achieve as comprehensive a result as possible. Originally 1,841 articles were identified using search terms including ‘sexual health, ‘HIV’, ‘STI’, ‘safe sex’, and ‘teenage pregnancy’. From this, articles were excluded if: less than 50% of the content related to issues of sexual health or BBV; they were published in Irish (Eire) editions of the newspapers (Ulster or Northern Irish editions were included); were short lead-ins that referred to a main story elsewhere in the same edition of the newspaper (if the main article also appeared in the sample); and/or were letters, advice, TV guides, sport, weather, obituaries and review pages. In total 1164 were excluded during filtering, leaving 677 articles for further consideration (see Figure 1).

Article headline and content tone were recorded as a means of assessing whether representations of sexual health and BBV issues were presented negatively, positively or were assessed as neutral or factual in tone. An article was rated as positive if the content focused on improvements and successes, outrage at wrongdoing or used optimistic, supportive and nonjudgemental language; in contrast, an article was assessed as negative if it highlighted failures, or portrayed a sense of hopelessness, fear or blame; and a rating of neutral or factual was given if no position was taken, if the article was largely factual, or if both positive and negative elements were present but neither was clearly more dominant than the other (mixed tone).

Here we present the analysis of a subgroup of the 677 articles originally identified in order to explore the negative framing of risk and responsibility in relation to gender. Articles were read independently by two researchers who worked in close collaboration, checking and validating each other’s coding to determine whether they met two inclusion criteria: that they were predominantly negative in tone and identified the gender of individuals focused on within the article. From the 677 articles identified, 435 were excluded as they were assessed as being predominantly positive or neutral/factual in tone and 92 were excluded as they did not identify gender (see Figure 1). A further 65 articles, which focused on two court cases prosecuting individuals for deliberate transmission of HIV, were excluded because they were considered to distort the sample. Therefore, 85 articles were included in the final sample for analysis.

A latent content analysis approach was taken, focusing on interpreting the underlying meaning of text, offering a more in-depth qualitative analysis of the data than examining manifest content alone [28]. All of the 85 articles were re-read multiple times before being coded in NVivo v.10 to help organise data thematically pulling together similarities and divergence in the descriptions of women, gay men and heterosexual men. Researchers worked in close collaboration to code articles, check interpretation, and used the constant comparative method and written summaries to verify the emerging findings [29, 30]. This manuscript adheres to the RATS guidelines for reporting qualitative studies.

## Results

Of the 85 articles, 40 were published in ‘mid-market’ newspapers, 24 published in ‘serious’ newspapers and 21 in ‘tabloid’ newspapers. Overall, 51 news articles focused on women and girls, 14 articles focused on men, and 20 articles included both sexes. Of the articles focused on men, seven had a particular focus on heterosexual men and seven on gay men. Even from this crude summary it was evident that in these negative articles there was a predominance of focus on women. Thematic and latent analysis of the data enabled grouping around issues of risk and responsibility for sexual health.

### Women, responsibility and men as risk to their health

A key theme in the articles was men being framed as a risk to women’s sexual health. Some articles referred to men pressuring women into not using condoms: *“…so much pressure from men not to wear a condom”* (*The Sun*, 26th Aug 2010); *“Doctors say young women are particularly vulnerable because they are sometimes being persuaded not to use condoms”* (*The Daily Mail*, 25th Aug 2010). In these extracts, men/boys were depicted as having the influence or power to ‘persuade’ women/girls to act in ways that might be risky, and teenage girls were frequently portrayed as being pressured to become involved in sexual relationships at an early age: *“…there is pressures on girls to get into sexual relationships way too early”* (*The Observer,* 29th Aug 2010). Risk not only included pregnancy, but also STIs. There were several articles reporting warnings of STI risks to females because men were reluctant to get tested: *“So a man could go on for years unaware that he has an infection he may be passing on to women, who are even less likely to develop symptoms”* (*The Mirror,* 7th Apr 2010); *“Young men don’t wear condoms and it appears it is young women who end up with the infection”* (Natika Halil of the Family Planning Association, *The Daily Mail*, 25th Aug 2010). Within the articles, it was also common for phrases such as “particularly amongst women” or “especially women” to follow statements highlighting increasing STI or teenage pregnancy rates. The framing of women as *“worst hit”* (Text extract, *The Sun*, 25th Aug 2010), “vulnerable” and particularly affected was common:

*“The Health Protection Agency, which compiled today's shocking figures, admitted they are "too high" - especially among under-25 s - and called for new safe-sex campaigns. It said girls are "particularly vulnerable"”* (*The Mirror*, 25th Aug 2010).

Some articles also reported on how the provision of contraception to teenage girls could place girls in a vulnerable position by encouraging boys to: *“bully them into sex”* (*The Daily Mail,* 25th Feb 2010). Another related theme in articles was the notion of part of a women’s role being to “resist” men’s advances. Such discourses tended to portray a power imbalance in sexual relationships between women and men, as highlighted by a quote from Nancy Mahon, Executive Director of the MAC Aids Fund, stating: *“British women feel they lack the power in their relationships to negotiate safe sex”* (*The Observer*, 28th Feb 2010). A number of articles argued that it was women who needed to take more responsibility for ensuring that they do not become infected with an STI or become pregnant (and teenage pregnancy articles rarely mentioned males). This focus on female responsibility was epitomised in one article commenting on the news of a women becoming pregnant to an infamous father of 14 children:

*“But idiots like MacDonald can only keep siring children because the women he beds have never heard the world contraception or are too dim to use it. MacDonald is undoubtedly a toe-rag but women have to bear some responsibility for what happens to them. A woman who doesn’t want to get pregnant does one of two things – she doesn’t have sex or she uses contraception”* (*News of the World,* 26th Sep 2010).

### Women, education and vulnerability

Articles repeatedly suggested that women and teenage girls in particular, lacked the skills and confidence to negotiate safe sex, further highlighting this sense of vulnerability concerning sexual health. A number of articles highlighted a need for women and girls to be re-alerted to the dangers of unprotected sex and hinted to significant gaps in HIV awareness and education and in sexual health knowledge more generally (*The Observer*, 28th Feb 2010). Articles that focused on HIV suggested that many British women did not think that this is a disease women are at risk from, with the findings of a study reporting that many women would never consider being tested: *“British women ‘in denial’ over growing HIV risk: False sense of security means females neglect to protect against virus”* (Headline, *The Observer*, 28th Feb 2010). Low levels of awareness were also mentioned in relation to young girls’ understandings of sex and contraception: “*research shows that there is a lot of confusion about contraception”* (*The Daily Record*, 22nd Mar 2010). However, a number of articles also focused on older women (described as a *“forgotten group”* in one article, text extract, *The Scotsman*, 9th Feb 2010) and their lack of awareness: *“Women aged over 35 who believe they can have unprotected sex because their fertility is on the wane are fuelling the demand for abortions, according to a sexual health charity”* (*The Scotsman*, 9th Feb 2010). There was also a focus on teenage girls not understanding or using contraception accurately as exemplified in this headline: *“Scores of teens having their third abortion; Girls ‘can’t do’ birth control”* (*The Mirror,* 14th Jun 2010). These discourses varied from girls lacking knowledge to discourses about girls being irresponsible in not ensuring safe sexual health and contraception practices. It was highlighted that there was a need for a greater focus on sex education: *“Starting sex education early, promoting strong female role models and continuing sex education programmes would help to encourage women to get tested and use protection”* (Dr Anke Ehrhardt, Director of the HIV Centre for Clinical & Behavioural Studies, *The Observer*, 28th Feb 2010). However, in contrast some were critical of sexual health education programmes and initiatives, arguing that they had failed to improve young women’s sexual health, citing evidence of rising rates of teenage pregnancy or STI’s to emphasise such ‘failures’. A number of articles went further than this, suggesting that sex education programmes and initiatives had not only failed, but had been harmful, contributing to women being at risk. For example, one reported that 16% of girls involved in a Teenage Pregnancy programme became pregnant – a higher proportion than in the general population, resulting in the initiative having *“a negative impact on the girls taking part”* (*The Daily Express,* 26th Aug 2010). It is of note that comparatively few articles focused on the need to educate men and boys in matter of sexual health compared to the focus on women and girls, suggesting that women are represented as being responsible even if not always in control.

### Men, responsibility and risk to their own health

Men were frequently portrayed as being more promiscuous and engaging in more risky sexual health behaviours than women. One published study was widely reported in the newspapers: *“Men are twice as likely as women to be promiscuous”* (*The Express*, 6th Apr 2010) and men “*…admitted to riskier behaviour 13 out of 100 men said they’d had unprotected sex with more than one person over the past five years, compared with just seven in 100 women”* (*The Mirror,* 7th Apr 2010)*.* Men were more likely to be presented as thoughtless and one article suggested that men were described as *“useless at thinking”* about their sexual health (*Sun,* 30th Apr 2010), while another article reported that STIs were seen by some men as a *“badge of honour”* (Genevieve Edwards from Terrence Higgins Trust, *The Sun*, 26th Aug 2010), and that males were governed by their *“primitive urges”* (*The Sun*, 26th Aug 2010). Articles also made reference to the common stereotype of men being reluctant to go to the doctors or seek medical help: *“…especially when it involves their manly bits”* (*The Mirror,* 7th Apr 2010) and several articles reported that despite having some of the highest levels of STIs, men usually waited until they had symptoms before getting tested and doing anything about their health. In contrast, just one article, reporting on recent research revealing that men are less likely to be screened and more likely to test positive for Chlamydia, drew attention to the lack of focus on male responsibility in safe sexual health behaviour: *“We have a duty to be responsible for not just our own sexual health but the consequences for others. That includes men too!”* (Text extract, *The Mirror,* 7th Apr 2010).

In articles describing infection rates among other at risk groups, gay men were often used as a bench mark against which rates were measured, for example: *“And men indulging in wifeswapping are suffering similar rates of infection as traditionally high risk groups such as gay men”* (*The Daily Record,* 24th Jun 2010). Gay men were also presented as being a health risk with increased rates of HIV and other STIs blamed on risky sexual behaviour, particularly among young gay men. Their behaviour was described as “*cavalier*” (*The Herald,* 30th Nov 2010), “*risky*” and “*unsafe*” (*The Guardian*, 7th Sep 2010). In addition, many articles made reference to gay men taking more risks with their sexual behaviour since the introduction of antiretroviral treatments for HIV. It was suggested that their widespread availability in recent years had contributed to many believing protection was not required, and that information about the real risks of HIV were no longer reaching or being understood by many men. One article mentioned that *“despite”* there being many prevention programmes and *“easily available testing facilities”* and *“supposedly broad public awareness of infection and possible routes of transmission”*, men who have sex with men continued to have high levels of HIV (*The Guardian,* 7th Sept 2010). Therefore although many resources are being targeted at and made easily available to gay men, it was suggested that many gay men were not taking responsibility for their sexual health and therefore continue to be at risk.

## Discussion

We conducted qualitative analysis of negatively toned newsprint coverage of sexual health topics published in the UK Press in 2010 to examine the representations of gender, risk and responsibility within these. It is evident that the way in which sexual health issues are framed and who is held responsible within the newsprint media could play an important role in shaping understanding and awareness of sexual health and illness. It has been suggested that the way in which sexual scenarios are portrayed in the media could be used by young people to fill gaps in understandings of sexual situations [31] and reinforce ‘norms’ they feel they should adhere to [24]. In our analysis, men were framed as a risk to women, and their sexual health, whilst women were presented as being at risk, but at the same time responsible for patrolling sexual encounters, organising contraception and preventing sexual ill health; reinforcing gender stereotypes.

Within feminist literature, men’s power over women is apparent and studies have indicated that the media adheres to this, portraying men stereotypically, as physically strong and mentally dominant, especially in interactions with women [32, 33]. Hedley et al. [32] found such gender stereotyping within their study of popular films, where women were presented as powerless and passive, whilst men were depicted as ‘bad’. Similarly, a study of sexual messages in music found that men typically demonstrated aggressive and dominant behaviour, while women tended to engage in sexual and subservient behaviour and were commonly the victims of implicit, explicit and/or aggressive sexual advances [34]. It seems that generally, studies have found a focus on men as ‘initiators’ or pursuers of sexual activity and on women as the ‘pursued’ [14, 22, 35]. This is supported by our analysis.

At the same time, the articles implied that women should be resisting and managing men’s sexual advances. This is a pattern found within other studies of television and magazine coverage, whereby girls are generally advised and encouraged to resist pressure to have sex [22, 36–38]. Thus, it seems that as well as female sexuality being coupled with being passive and vulnerable, it is also associated with responsibility [39]. This is further evidence that there is a sexual double standard, where the sexual activity of young men is encouraged and tolerated, while for young women, it is discouraged, controlled, restricted, and subjected to condemnation and sanctioning if norms are violated [14, 40–43].

Our analysis highlighted a further contradiction: as well as being at risk and needing protection, women were presented as having responsibility for safer sex. Generally, boys and men were invisible in reports on teenage pregnancy, abortion and contraception within our sample, and there was little or no acknowledgement of male responsibility for contraception and safer sex practices. A number of other studies analysing gender roles within media coverage found similar commonplace framings of unsafe sex and its consequences as ‘women’s problems’ or concerns [22, 24, 44]. Conversely, conversations about sex amongst male characters on television have been found to centre on boasting about sexual prowess and pride in sexual performance with little consideration of risk or consequences [22]. Attributing ‘blame’ or responsibility along gender stereotypes encourages the belief that the problem of, and consequently the solution to, prevention lies with the behaviours of a particular group, in this case women. It seems that burdening women with responsibility enables men’s lack of involvement and ignorance in relation to their own and their partners’ health and very much discourages engagement and equality in sexual health promotion [22, 25].

Attention has been drawn to the typical solution to male pressure and sexual relationships within sexuality education in the US: the encouraging and teaching of girls to abstain [45]. Such a focus (often advocated by moral conservative and religious groups) was a common feature within articles on sex education in our sample. Whilst a few articles highlighted the need for better sex education for women and girls to enable them to make informed choices, most focused on the failures of such programmes, presenting them as contributing to women and girls being at risk, and blaming them for encouraging sexual activity, experimentation, unprotected sex and promiscuity. Our findings are consistent with other studies carried out in this area [46]. In particular, a number of studies analysing the representation of the HPV vaccination in newsprint media found that, despite relatively positive descriptions of vaccination, there was a focus on infection rates amongst women and on risky sexual behaviours as a consequence of vaccination [15, 47]. Similarly, a number of articles portrayed the provision of emergency contraception as particularly ‘risky’ and responsible for promoting promiscuity, despite evidence to the contrary [48]. There seemed to be a paternalistic ideology present within coverage, with a focus on the need to protect young girls from such policies/initiatives and prevent the sexual experimentation they would “inevitably” encourage. Focusing on discouragement and abstinence over access to information and contraception may work to reinforce traditional, stereotypical gender roles, and thus fail to acknowledge or challenge gender inequality [49].

The exception to the lack of focus on men, was in relation to articles on gay men, who were presented as *a* risk, *at* risk, and also failing to be responsible. There were suggestions that the widespread availability of antiretrovirals for HIV treatment had resulted in ‘cavalier attitudes’ amongst gay men. A number of UK community-based studies have reported increasing rates of sexual risk behaviour amongst gay men [50, 51], and some have questioned the link between awareness or use of antiretrovirals and high risk sexual behaviours [52]. However, studies have found little association between the two [52, 53]. Also, we found that gay men were often not the main focus of articles, but were instead included as a bench mark against which rates in other populations were measured (the issue of STI infection among gay men in some ways was taken for granted and assumed to be widely known).

The findings of this research needs to be considered in light of its limitations. Firstly, this study included only newsprint media and is therefore not representative of the broader media’s role in the portrayal of sexual health and BBV issues. However, newsprint media does provide a widely used data source, and although there is a move towards digital news formats, there is no evidence that the reporting in them is vastly different. Also, the one-year time period was short and only represents a ‘snapshot’; preventing the measuring of trends over time. Despite such limitations, this piece of research comes from the first large-scale exploration of UK national newsprint media representation of all sexual health and BBV topics since the 1990s and provides valuable insights into the newsprint media’s role in shaping understandings of gender roles in relation to risk and responsibility in sexual health.

## Conclusions

It is important to acknowledge that our sample consisted of articles deemed to be negative in tone and sexual health organisations have made progress in working to quell the negative tone of media reporting around sexual health, whilst positively influencing public debate [46]. However, the reinforcement of stereotypes and unbalanced attribution of responsibility along gender lines found within our analysis has implications for sexual ill health prevention and could continue to reinforce a negative culture around sex, relationships and sexual health in the UK. The importance of raising awareness of gender conflicts, promoting sexual and reproductive rights and discussing ethical issues relating to respect for autonomy in decision-making has been stressed [54, 55]. The media should be encouraged to portray sexual health issues in a positive and balanced way to encourage ‘collective responsibility’ for sexual health and promote healthy sexual attitudes. To facilitate this, such positive portrayals should be employed by sexual health professionals and public health advocates in their future communication strategies. This will be key if the Scottish Government is to achieve its desired aim of creating a society and culture in which the attitudes towards sexual health are positive, non-stigmatising and supportive [2]. Further research could focus on how influential media messaging is by shedding light on the possible impact of such negatively toned messaging (and also positively toned) on individual and community perceptions, attitudes, beliefs and behaviours in relation to risk, responsibility and sexual health.

## References

[1] Department of Health: **A framework for sexual health improvement in England.** [https://www.gov.uk/government/publications/a-framework-for-sexual-health-improvement-in-england] 2010. Accessed 07/13

[2] Scottish Government: **The sexual health and blood borne virus framework 2011–15.** [http://www.scotland.gov.uk/Publications/2011/08/24085708/16] 2011. Accessed 07/13

[3] Kline KN, Thompson TL, Dorsey AM, Miller KI, Parrott R (2003). Popular Media and Health: Images, Effects, and Institutions. Handbook of Health Communication.

[4] Seale C (2003). Health and media: an overview. Sociol Health Ill.

[5] Entman RM (1993). Framing: towards clarification of a fractured paradigm. J Commun.

[6] McCombs ME, Shaw DL (1972). The agenda-setting function of mass media. Public Opin Quart.

[7] Iyengar S (1990). Framing responsibility for political issues: the case of poverty. Polit Behav.

[8] Pan Z, Kosicki G (1993). Framing analysis: an approach to news discourse. Polit Commun.

[9] Sutton MJ, Brown JD, Wilson KM, Klein JD, Brown JD, Steele JR, Walsh-Childers K (2002). Shaking the Tree of Knowledge for Forbidden Fruit: Where Adolescents Learn About Sexuality and Contraception. Sexual Teens, Sexual Media. Investigating Media’s Influence on Adolescent Sexuality.

[10] L’Engle KL, Brown JD, Kenneavy K (2006). The mass media are an important context for adolescents’ sexual behavior. J Adolesc Health.

[11] Brown JD, L’Engle KL, Pardun CJ, Guo G, Kenneavy K, Jackson C (2006). Sexy media matter: exposure to sexual content in music, movies, television, and magazines predicts black and white adolescents’ sexual behavior. Pediatrics.

[12] Collins RL, Elliott MN, Berry SH, Kanouse DE, Kunkel D, Hunter SB, Miu A (2004). Watching sex on television predicts adolescent initiation of sexual behavior. Pediatrics.

[13] Bandura A (1977). Social Learning Theory.

[14] Aubrey JS (2004). Sex and punishment: an examination of sexual consequences and the sexual double standard in teen programming. Sex Roles.

[15] Hilton S, Hunt K, Langan M, Bedford H, Petticrew M (2010). Newsprint media representations of the introduction of the HPV vaccination programme for cervical cancer prevention in the UK (2005–2008). Soc Sci Med.

[16] Bryant J, Zillmann D (2004). Media effects: advances in theory and research.

[17] Burton G: **Media and Society: Critical Perspectives.** Maidenhead, England: Open University Press; 2005

[18] Kline KN (2006). A decade of research on health content in the media: the focus on health challenges and sociocultural context and attendant informational and ideological problems. J Health Commun.

[19] Sharf BF, Freimuth VS (1993). The construction of illness on entertainment television: coping with cancer on thirtysomething. Health Comm.

[20] Kunkel D, Biely E, Eyal K, Cope-Farrar K, Donnerstein E, Fandrich R: **Sex on TV 3: A biennial report of the Kaiser Family Foundation.** Menlo Park, CA: Henry J. Kaiser Family Foundation; 2003

[21] Fisher DA, Hill DL, Grube JW, Gruber EL (2004). Sex on American television: an analysis across program genres and network types. J Broadcast Electron.

[22] Batchelor SA, Kitzinger J, Burtney E (2004). Representing young people’s sexuality in the ‘youth’ media. Health Educ Res.

[23] Cantrell EA: **No Angel: An Analysis of Media Coverage of Nadja Benaissa in the U.K., U.S. and Germany.** Thesis, Georgia State University; 2011 http://scholarworks.gsu.edu/communication_theses/84/

[24] Hust SJT, Brown JD, L’Engle KL (2008). Boys will be boys and girls better be prepared: an analysis of the rare sexual health messages in young adolescents’ media. Mass Comunn Soc.

[25] Quinlan MM, Bute JJ (2013). ‘Where are all the men?’ A post-structural feminist analysis of a university’s sexual health seminar. Sex Educ.

[26] Martin S, Hilton S, McDaid LM (2013). United Kingdom newsprint media reporting on sexual health and blood-borne viruses in 2010. Sex Health.

[27] Hilton S, Hunt K (2011). UK newspapers’ representations of the 2009–10 outbreak of swine flu: one health scare not over-hyped by the media?. J Epidemiol Community Health.

[28] Downe-Wamboldt B (1992). Content analysis: method, applications, and issues. Health Care Women Int.

[29] Glaser BG, Strauss AL (1967). The Discovery of Grounded Theory.

[30] Lincoln YS, Guba EG (1985). Naturalistic Inquiry.

[31] Brown JD, Steele JR (1995). Sex and the Mass Media.

[32] Hedley M (1994). The presentation of gendered conflict in popular movies: affective stereotypes, cultural sentiments, and men’s motivation. Sex Roles.

[33] Vigorito AJ, Curry TJ (1998). Marketing masculinity: gender identity and popular magazines. Sex Roles.

[34] Sommers-Flanagan R, Sommers-Flanagan J, Davis B (1993). What’s happening on music television - a gender-role content analysis. Sex Roles.

[35] Kunkel D, Cope-Farrar K, Biely E, Maynard Farinola WJ, Donnerstein E (2001). Sex on TV (2) A Biennial Report to the Kaiser Family Foundation.

[36] Wray J, Steele JR, Brown JD, Steele JR, Walsch-Childers K (2001). What It Means To Be A Girl: Teen Girl Magazines. Sexual Teens, Sexual Media.

[37] Tolman DL (1994). Doing desire: adolescent girls’ struggles for/with sexuality. Gender Soc.

[38] Tolman DL, Johnson NG, Roberts MC, Worell J (1999). Female Adolescent Sexuality in Relational Context: Beyond Sexual Decision-Making. Beyond Appearance: A new Look at Adolescent Girls.

[39] Carpenter LM (1998). From girls into women: scripts for sexuality and romance in Seventeen magazine,1974–1994. J Sex Res.

[40] Jessor SL, Jessor R (1975). Transition from virginity to non-virginity among youth: a social–psychological study over time. Dev Psychol.

[41] MacCorquodale P, McKinney K, Sprecher S (1989). Gender and Sexual Behavior. Human Sexuality: The Societal and Interpersonal Context.

[42] Muehlenhard CL (1988). “Nice women” don’t say yes and “real men” don’t say no: How miscommunication and the double standard can cause sexual problems. Women Ther.

[43] Walsh-Childers K, Brown JD, Greenberg BS, Brown JD, Buerkel-Rothfuss N (1993). Adolescents’ Acceptance of Sex-Role Stereotypes and Television Viewing. Media, Sex, and the Adolescent.

[44] Bute JJ, Harter LM, Kirby EL, Thompson M, Hayden S, O’Brien Hallstein DL (2010). Politicizing Personal Choices? The Storying of Age-Related Infertility in Public Discourses. Contemplating Maternity in an Era of Choice: Explorations into Discourses of Reproduction.

[45] Tolman D, Hirschman C, Impett E (2005). There’s More to the Story: The place of qualitative research on female adolescent sexuality in policy making. Sexuality Res Policy Stud.

[46] Kingori P, Wellings K, French R, Kane R, Gerressu M, Stephenson J (2004). Sex and relationship education and the media: an analysis of national and regional newspaper coverage in England. Sex Educ.

[47] Forster A, Wardle J, Stephenson J, Waller J (2010). Passport to promiscuity or lifesaver: press coverage of HPV vaccination and risky sexual behavior. J Health Commun.

[48] Camp SL, Wilkerson DS, Raine TR (2003). The benefits and risks of over-the-counter availability of levonorgestrel emergency contraception. Contraception.

[49] Decarie M (2005). Threatening Disaster, Promising Salvation: An Analysis of Discourses in Abstinence-Only Sex Education.

[50] Mercer CH, Fenton KA, Copas AJ, Wellings K, Erens B, McManus S, Nanchahal K, Macdowall W, Johnson AM (2004). Increasing prevalence of male homosexual partnerships and practices in Britain 1990–2000: evidence from national probability surveys. AIDS.

[51] Knussen C, Flowers P, McDaid LM, Hart GJ (2011). HIV-related sexual risk behaviour between 1996 and 2008, according to age, among men who have sex with men (Scotland). Sex Transm Infect.

[52] Speakman A, Rodger A, Phillips AN, Gilson R, Johnson M, Fisher M, Ed W, Anderson J, O’Connell R, Lascar M, Aderogba K, Edwards S, McDonnell J, Perry N, Sherr L, Collins S, Hart G, Johnson AM, Miners A, Elford J, Geretti AM, Burman WJ, Lampe FC (2013). The ‘Antiretrovirals, Sexual Transmission Risk and Attitudes’ (ASTRA) Study. Design, methods and participant characteristics. PLoS One.

[53] Kozal MJ, Amico KR, Chiarella J, Schreibman T, Cornman D, Fisher W, Fisher J, Friedland G (2004). Antiretroviral resistance and high-risk transmission behavior among HIV-positive patients in clinical care. AIDS.

[54] Schenk KD (2003). Emergency contraception: lessons learned from the UK. J Fam Plann Reprod Health Care.

[55] Cook RJ, Dicken BM (2003). Access to emergency contraception. J Obstet Gynaecol Can.

[CR56] The pre-publication history for this paper can be accessed here:http://www.biomedcentral.com/1471-2458/14/792/prepub

